# Performances of Four *Helicobacter pylori* Serological Detection Kits Using Stool Antigen Test as Gold Standard

**DOI:** 10.1371/journal.pone.0163834

**Published:** 2016-10-13

**Authors:** Susheela D. Biranjia-Hurdoyal, Sharmila P. Seetulsingh-Goorah

**Affiliations:** 1 Department of Health Sciences, University of Mauritius, Moka, Mauritius; 2 University Technology of Mauritius, Moka, Mauritius; George Mason University, UNITED STATES

## Abstract

The aim was to determine the performances of four *Helicobacter pylori* serological detection kits in different target groups, using Amplified IDEIA^™^ Hp StAR^™^ as gold standard. Kits studied were Rapid Immunochromatoghraphic Hexagon, Helicoblot 2.1, an EIA IgG kit and EIA IgA kit. **Methods:** Stool and blood samples were collected from 162 apparently healthy participants (control) and 60 Type 2 diabetes mellitus (T2DM) patients. **Results:** The performances of the four serological detection kits were found to be affected by gender, age, health status and ethnicity of the participants. In the control group, the Helicoblot 2.1 kit had the best performance (AUC = 0.85; p<0.05, accuracy = 86.4%), followed by EIA IgG (AUC = 0.75; p<0.05, accuracy = 75.2%). The Rapid Hexagon and EIA IgA kits had relatively poor performances. In the T2DM subgroup, the kits H2.1 and EIA IgG had best performances, with accuracies of 96.5% and 93.1% respectively. The performance of EIA IgG improved with adjustment of its cut-off value. **Conclusion:** The performances of the detection kits were affected by various factors which should be taken into consideration.

## Introduction

*H*. *pylori* has been associated with several gastrointestinal diseases, such as gastritis, gastric ulcer/duodenal ulcer and mucosa associated lymphoid tissue lymphoma [1–3] and extra-gastrointestinal diseases, such as iron deficient anaemia [4], idiopathic thrombocytopenic purpura [5], non-communicable diseases, including diabetes mellitus and cardiovascular diseases [6,7]. Several invasive diagnostic methods, such as endoscopy (CLO tests, histology, culture) and non-invasive methods, such as serological tests, stool antigen detections, urea breath test have been used to determine the *H*. *pylori* infection status [8–10]. The performances of serological tests have been found to be affected by factors such as type of samples, population under study, strain of *H*. *pylori* harboured by the patient and strain used to manufacture the detection kit [8,11–13].

In absence of invasive methods, the Maastricht IV/Florence Consensus Report and the Second Asia-Pacific Consensus guidelines for *H*. *pylori* infection, have recommended urea breath test and EIA stool monoclonal antigen tests as the preferred methods of detection of *H*. *pylori* [14,15]. Many clinical settings and laboratories do not have the infrastructure and facilities to carry out urea breath test. Therefore, non-invasive tests, such as serological test and stool antigen detection have been mostly used and reported. However, stool antigen tests and urea breath test cannot be used for patients on antibiotics, anti-secretory drugs and those suffering from ulcer bleeding [14]. Japan and South Korea have recommended IgG serological detection as one of their preferred detection method for initial diagnosis [16].

Several studies have investigated the possible role of *H*. *pylori* in diseases on the basis of the prevalence of the bacterium in the population. Given, the accuracy of detection kits vary between populations, conflicting data on the role of the bacterium in diseases have been reported [17–19]. Therefore, it is important to validate and determine the detection kit with the best performance in a given population, prior to determining the prevalence of *H*. *pylori* and its exact role in diseases. It has been recommended that all detection tests should be used after appropriate validation in the local population [14–15].

In Mauritius, several types of serological kits and stool antigen kits are used to determine *H*. *pylori* infection status. No study has previously validated and reported any *H*. *pylori* detection kit among Mauritians. Therefore, in this study, using the same study population, we have evaluated four different serological detection kits, Rapid Immunochromatoghraphic Hexagon *H*. *pylori* by Human (Rapid Hx), HELICO BLOT 2.1 by MP Diagnostics (H 2.1), Premier^™^ *H*. *pylori* by Meridian Bioscience, Inc (EIA IgG) and *H*. *pylori* IgA ELISA by DSL (EIA IgA), by comparing their performances with a stool monoclonal antigen kit, Amplified IDEIA^™^ Hp StAR^™^ by Dakocytomation (Hp StAR). The various factors which could potentially affect the performances of the serological detection kits were also investigated, which included age, health status, gender and ethnicity.

## Materials and Methods

### Study population

A total of 285 participants aged between 30–65 years were interviewed, out of which 222 individuals satisfied the inclusion criteria and were recruited with the help of a questionnaire. The participants were never subjected to eradication regimen for *H*. *pylori* or had not received proton pump inhibitors and antibiotics during the previous month. The control group consisted of 162 apparently healthy participants, including 88 females and 74 males, who did not have any stomach problems associated with *H*. *pylori* infection and were not suffering from any health conditions which required medical assistance. The second group included 30 females and 30 males who were suffering from type 2 diabetes mellitus (T2DM). The participants were recruited from the ambulatory general public from various regions of Mauritius. The study was approved by the University of Mauritius Research Ethics Committee, Mauritius and written consent was obtained from all participants.

### Samples

Each participant provided a blood and stool sample which were coded and processed within one week. The presence of *H*. *pylori* antibodies was detected using Rapid Hx, H 2.1, EIA IgG and EIA IgA. The *H*. *pylori* antigen was detected in the stool samples by using the Hp StAR.

### Definition of *H*. *pylori* status

The *H*. *pylori* status was defined as positive when the stool antigen test was positive. Hp StAR detection kit was used as the gold standard to determine the performances of the four serological kits in the control and T2DM participants.

### Detection kits

All tests were performed and results read according to manufacturer’s instructions. Rapid Hx detected qualitatively the presence of *H*. *pylori* IgG, IgA and IgM in human whole blood, serum or plasma. The control line consisted of anti *H*. *pylori* antibodies and the test line that of *H*. *pylori* antigen. The mobile phase employed *H*. *pylori* antigen conjugated to colloidal gold. When the test strip was subjected to the sample, the *H*. *pylori* antibodies formed a complex with the dye conjugate, which then bound to the specific antigen present in the test line. These reactions were seen as colour change in the test and control line. The H 2.1 consisted of *H*. *pylori* lysate which detected specific IgG to the various proteins of the bacterium in human plasma or serum. The kit also has a recombinant antigen, known as the current infection marker (CIM), which has a high predictive value for the indication of current infection status. EIA IgG kit detected IgG to *H*. *pylori* in serum samples by binding to the sonicated *H*. *pylori* cell lysate coated on the well surface while EIA IgA kit determined IgA to *H*. *pylori* by binding to the inactivated and purified *H*. *pylori* antigens on the well surface. Hp StAR kit detected *H*. *pylori* antigens in stool samples which bind specifically to the surface of the wells of the microplate, which have been coated with monoclonal antibodies specific to *H*. *pylori*. All the EIA kits were processed and results were read photometrically at 450nm.

### Assay validation

Precision of the diagnostic methods was determined by the intra and inter-assay co-efficient of variability (CV). For the intra-assay CV, repeated assays of four samples were done within a single process, every time the EIA kits were used, while for the inter-assay CV, four samples were repeatedly analysed in the consecutive batches of the EIA runs. For the precision of the Rapid Hx and H2.1, the results were read by two observers. The intra-assay CV was ≤ 6.7% while the inter-assay CV was ≤ 9.70%.

### Data analysis

The diagnostic performances of the kits have been reported as sensitivity (sen), specificity (spec), accuracy (acc), Kappa Cohen Coefficient (*k*) and area under ROC curve (AUC). The *k* value determined the agreement between the serological kits and the gold standard. A *k* value of <0.20 was read as poor agreement, 0.21–0.40 as fair, 0.41–0.60 as moderate, 0.61–0.80 as good agreement and more than 0.81 as very good agreement. The results of AUC was read as excellent if the area was between 0.9 to1.0, good for 0.8–0.9, fair for 0.7–0.8, poor for 0.6–0.7 and fail for an area 0.6–0.5. The AUC was also read as statistically significant, if the AUC value was greater than 0.5 and p<0.05. The statistical analysis was done using SPSS v.16.0 (SPSS Inc, California, USA) and p value < 0.05 was established as significant.

## Results

The percent positive to *H*. *pylori* for each subgroup is summarised in Table 1.

**Table 1:** Percent positive of *H*. *pylori* in apparently healthy and T2DM participants.

Target group	n	Percent positive of *H*. *pylori* using				
		Rapid Hx	H 2.1	EIA IgG	EIA IgA	Hp StAR
AH F	88	71.6	60.0	51.1	37.5	44.3
AH M	74	68.9	79.2	77.0	54.1	68.9
All AH ppts	162	70.4	68.9	63.0	45.1	55.6
T2DM F	30	58.6	63.5	63.3	45.5	53.6
T2DM M	30	76.0	71.2	76.7	66.7	73.3
All T2DM	60	66.7	67.3	70.0	55.0	63.8

n-sample size; F-females; M-males; AH-apparently healthy; ppts-participants; T2DM-Type 2 diabetes mellitus.

The performances of the 4 serological kits were determined usingHp StAR (Tables 2 and 3). All the four serological kits had statistically significant AUC values in both groups. However, the overall performances of the four serological kits were better in T2DM than in the control population.

**Table 2:** Performances of Rapid Hx and H2.1 kits using Hp StAR as gold standard.

Target groups	Test performance of Rapid Hx					Test performance of H2.1				
	Sen	Spec	AUC	Acc	K	Sen	Spec	AUC	Acc	K
AH F	89.7	42.9	0.66 p<0.05	63.6	0.31	100	80	0.90 p<0.05	88.6	0.78
AH M	78.4	52.2	0.65 p<0.05	70.3	0.31	98.0	52.2	0.75 p<0.05	83.8	0.57
All AH	83.3	45.8	0.65 p<0.05	66.7	0.30	98.9	70.8	0.85 p<0.05	86.4	0.72
T2DM F	85.7	61.5	0.73 p<0.05	74.1	0.48	100	92.3	0.94 p<0.05	96.4	0.93
T2DM M	100	75	0.92 p<0.05	92.3	0.81	100	87.5	0.92 p<0.05	96.7	0.91
All T2DM	93.7	70	0.82 p<0.05	84.6	0.66	100	90.5	0.93 p<0.05	96.5	0.92

SEN: sensitivity; SPE: Specificity; AUC: area under curve; p value of AUC- indicates whether the AUC obtained is statistically different from AUC value 0.5; Acc: accuracy; AH- apparently healthy; T2DM-Type 2 diabetes mellitus.

**Table 3:** Performances of EIA IgG and EIA IgA kits using Hp StAR as gold standard.

Target groups	Test performance of EIA IgG						Test performance of EIA IgA				
	cut off value	Sen	Spec	AUC	Acc	K	Sen	Spec	AUC	Acc	K
AH Females (n = 88)	0.12	100	57.1	0.79 p<0.05	76.1	0.54	64.1	65.3	0.68 p<0.05	64.8	0.29
	0.25	97.4	83.7	0.89 p<0.05	89.8	0.80					
AH males (n = 74)	0.12	100	35	0.67 p<0.05	74.5	0.42	64.7	52.2	0.63 p = 0.07	60.8	0.16
	0.25	94.1	60.9	0.78 p<0.05	83.7	0.59					
AH ppts (n = 162)	0.12	100	49.2	0.75 p<0.05	75.2	0.53	64.4	61.1	0.67 p<0.05	63	0.25
	0.25	94.4	76.4	0.85 P<0.05	86.4	0.72					
T2DM Females (n = 30)	0.12	100	76.9	0.89 p<0.05	89.3	0.78	81.8	100	0.86 p<0.05	90	0.80
	0.25	100	76.9	0.89 p<0.05	89.3	0.78					
T2DM males (n = 30)	0.12	100	87.5	0.92 P<0.05	96.7	0.91	83.3	50	0.74 p = 0.11	72.2	0.35
	0.25	100	87.5	0.92 p<0.05	96.7	0.91					
All T2DM (n = 60)	0.12	100	81	0.91 p<0.05	93.1	0.84	82.6	80	0.78 p<0.05	81.6	0.62
	0.25	100	81	0.91 P<0.05	93.1	0.84					

SEN: sensitivity; SPE: Specificity; AUC: area under curve; p value of AUC- indicates whether the AUC obtained is statistically different from AUC value 0.5; Acc: accuracy; AH- apparently healthy; T2DM-Type 2 diabetes mellitus.

Among the T2DM participants, the H2.1 had the best performance, followed by EIA IgG, Rapid Hx and lastly EIA IgA. Furthermore, among the T2DM females, EIA IgA had better performance than Rapid Hx, whereas among the T2DM males, performance was better using the Rapid Hx than EIA IgA kit. The OD cut off value 0.120 recommended by the manufacturer for the EIA IgG was not found to be appropriate for the apparently healthy Mauritian population, as an increase in false positive results was noted. The cut-off value was redefined to 0.25 for the local population using Hp StAR as gold standard (Table 3). The performance of EIA IgA was not affected by OD cut off value and therefore, the recommended OD cut off value by the manufacturer was maintained.

IgA was not detected in 43 (26.5%) of the controls who were IgG positive. It was noted that as the percentage of samples with discrepancies in IgG/IgA test results increased, the accuracy and the AUC values of the Rapid Hx decreased, hence lowering its performances (Table 4).

**Table 4:** Effect of discrepancies in IgA detected with EIA IgA and IgG detected with H2.1 on the performance of Rapid Hx.

Target groups	IgA+/IgG- n (%)	IgA-/IgG+ n (%)	Samples with discrepancies n (%)	Performance of Rapid Hx
AH	15 (9.26)	43 (26.5)	58 (35.1)	Acc = 66.7; AUC = 0.65; p<0.05
T2DM	3 (5.0%)	6 (10.0%)	9 (15.0%)	Acc = 84.6; AUC = 0.82; p<0.05

AH-apparently healthy; T2DM-Type 2 diabetes mellitus; Acc-accuracy; AUC-area under ROC curve.

Among the apparently healthy participants, the performance of EIA IgG was lower at age 46–55 years, compared to 30–45 years and 56–65 years, whereas the overall performance of EIA IgA increased with age. The performances of the four serological kits were better among AH females compared to AH males. Disease status also affected performances, as the overall performances of the four serological kits were better in T2DM than the control population. The H2.1, EIA IgG and EIA IgA serological kits also had better performances among the Mauritian of Indian origin compared to the Mauritian of African origin.

Given, that H2.1 was found to have the best performance next to Hp StAR, the ability of CIM to detect active infection was also investigated (Table 5).

**Table 5:** The performance of CIM using Hp StAR as gold standard.

Target groups	n	SEN	SPE	Acc	AUC	K
AH females	88	84.6	85.7	85.2	0.85; p<0.05	0.70
AH males	74	88.3	60.9	79.7	0.75; p<0.05	0.51
AH ppts	162	86.7	77.8	82.7	0.82; p<0.05	0.65
T2DM females	30	80.0	100	89.3	0.90; p<0.05	0.79
T2DM males	30	72.7	100	80.0	0.86; p<0.05	0.59
T2DM ppts	60	75.7	100	84.5	0.88; p<0.05	0.69

n- sample size; SEN-sensitivity; SPE-Specificity; Acc- acccuracy of kit; AUC-area under ROC curve; p value of AUC- indicates whether the AUC obtained is statistically different from AUC value 0.5; AH-apparently healthy; T2DM-Type 2 Diabetes Mellitus

## Discussion

This study has compared the performances of a rapid test, western blot, EIA IgG and EIA IgA in a single study using same the study population. Our study provides important information on serological kits which could reliably detect *H*. *pylori* infection among Mauritians. The H2.1 was found to be the best serological test for detecting *H*. *pylori* infection in Mauritian population. The AUC ranged from good to excellent in all the subgroups, which was in agreement with previous studies which reported sensitivities between 80% to 98.6% and specificities 87.1% to 100% [20–22]. The sensitivity of the H2.1 in all the subgroups was excellent. However, a decrease in specificity was noted, which could be due to cross-reactive antigens [9] and loss of the three-dimensional conformation of the IgG antibodies [23]. Furthermore, it has been suggested that a sensitive test might be used when the prevalence of *Helicobacter pylori* is high and a specific test might be chosen, when the prevalence of disease is low [24]

The performances of the detection kits were found to be affected by several factors, such as discrepancies in IgA and IgG response by the host, age, gender, health status and ethnicity. The overall performances of the four serological detection kits were better in the T2DM compared to the control. The reason could be the genetic variability of *H*. *pylori* strains in the various subgroups. Previous studies have associated the high level of genetic differences in the CagA and VacA genes with diseases, such as mucosa associated lymphoid tissue lymphoma, gastric cancer and peptic ulcer [25–27]. It should be noted that no molecular study has been done to compare the genetic variability of the bacterium between apparently healthy individuals and those suffering from non-communicable disease, such as diabetes mellitus. Furthermore, this is the first study to report the difference in performances of various detection kits in healthy individuals and patients suffering from a non-communicable disease, such as T2DM.

The EIA IgG had an overall better performance as compared to EIA IgA and Rapid Hx. Several researchers have also reported better performances of EIA IgG than EIA IgA kits [28,29]. It has been reported that 2–7% of patients produced IgA in the absence of IgG. Furthermore, IgA remained undetected in 33% of *H*. *pylori* infected individuals who were positive by IgG tests [30]. In this study, it was noted that 14.3% of males and 15.9% of females were positive to IgG but negative to IgA. As the percentage of samples with discrepancies in IgG and IgA detection increased, the AUC value of Rapid Hx decreased, hence, indicating lower sensitivities and specificities

Furthermore, the performance of EIA IgG was found to be affected by the age of the participants and the OD cut-off value recommended by the manufacturer. The re-adjusted OD cut-off value from 0.120 to 0.250 for the local population increased the AUC value, sensitivity and specificity. Previous studies have also recommended local validation by optimising cut-off values for serological detection kits [31]. Moreover, a significant decrease in specificity, accuracy and kappa value was noted among apparently healthy participants aged 46–55 years. The decrease in performance of EIA IgG in females aged 46–55 years might be due menopause, a stage at which there is low production of oestrogens, sex steroid hormones, which are known to enhance humoural immune responses. Furthermore, it has also been reported that males generally have lower immune responses than females, as testosterone could suppress the activity of immune cells [32].

The presence or absence of Vac A and Cag A was determined from the results of the H2.1. It was noted that as the prevalence of Vac A negative strains increased in the population, the percentage of samples read as negative by Rapid Hx also increased and hence, lowering its performance. Thus, indicating that most probably the Rapid Hx kit was not designed to detect all strains of *H*. *pylori*. Difference in the performances of detection kits in various ethnic groups has also been previously reported [33]. However, no study has compared individuals of Indian and African origin from the same country.

In absence of invasive detection methods and Hp StAR, CIM could be recommended to be used to determine active infections in both apparently healthy and T2DM individuals aged between 30–65 years. However, it has been previously reported that CIM band could not differentiate between past and on-going infection [22].

### Limitation

In this study, invasive methods were not used to determine *H*. *pylori* status because of ethical issues. Furthermore, urea breath test is not practiced in Mauritius. Therefore, monoclonal EIA stool antigen test was used as the gold standard.

### Conclusions

Different kits have different performances in the same population and the same kit has different performance in different population. It is vital for every country to validate its *H*. *pylori* detection kits to be used for its population. Both H2.1 and EIA IgG had similar and best performances and could be recommended to be used in the local Mauritian population. The OD cut off value of EIA IgG should be revised to 0.250 for the Mauritian population and could be used for individuals aged less than 45 years of age. The kit of choice would be H2.1 in all age groups. The performances of the serological detection kits were found to be affected by the antibody response (IgG or IgA), age, gender, health status and ethnicity of the host.
